# Supplementary material for: Biceps Tenodesis Better Improves the Shoulder Function Compared with Tenotomy for Long Head of the Biceps Tendon Lesions: A Meta-Analysis of Randomised Controlled Trials
Source: J Clin Med. 2023 Feb 22;12(5):1754. doi: 10.3390/jcm12051754 (PMC10003204; doi:10.3390/jcm12051754)

Figure S1: meta-analysis results of the improvement of ASES

Study or Subgroup	Tenotomy Mean	Tenotomy SD	Tenotomy Total	Tenodesis Mean	Tenodesis SD	Tenodesis Total	Weight	Mean Difference IV, Random, 95% CI	Year
MacDonald 2020	35	16.93	52	31.2	20.03	48	39.6%	3.80 [-3.50, 11.10]	2020
Hufeland 2019	31	20.5	11	46.5	13.26	9	21.9%	-15.50 [-30.39, -0.61]	2019
Oh 2016	42.72	17.87	27	39.15	10.61	31	38.5%	3.57 [-4.14, 11.28]	2016
**Total (95% CI)**			**90**			**88**	**100.0%**	**-0.51 [-9.58, 8.56]**	

Heterogeneity: Tau² = 40.14; Chi² = 5.66, df = 2 (P = 0.06); I² = 65%
Test for overall effect: Z = 0.11 (P = 0.91)

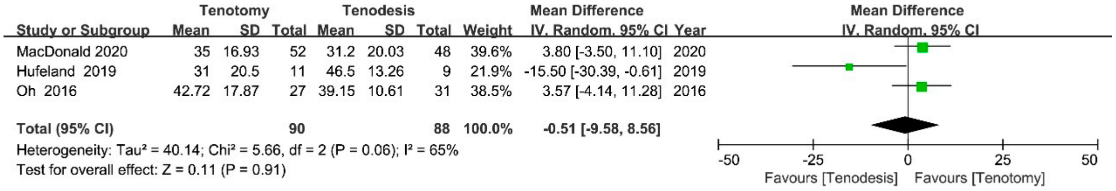

Figure S2: meta-analysis results of the improvement of SST

Study or Subgroup	Tenotomy Mean	Tenotomy SD	Tenotomy Total	Tenodesis Mean	Tenodesis SD	Tenodesis Total	Weight	Mean Difference IV, Fixed, 95% CI	Year
Hufeland 2019	5.2	2.49	11	5.3	2.26	9	3.9%	-0.10 [-2.18, 1.98]	2019
Mardani-Kivi 2018	7.07	1.44	29	7.42	1.12	33	40.5%	-0.35 [-1.00, 0.30]	2018
De Carli 2012	6.2	1.13	30	7.3	1.14	35	55.6%	-1.10 [-1.65, -0.55]	2012
**Total (95% CI)**			**70**			**77**	**100.0%**	**-0.76 [-1.17, -0.34]**	

Heterogeneity: Chi² = 3.37, df = 2 (P = 0.19); I² = 41%
Test for overall effect: Z = 3.60 (P = 0.0003)

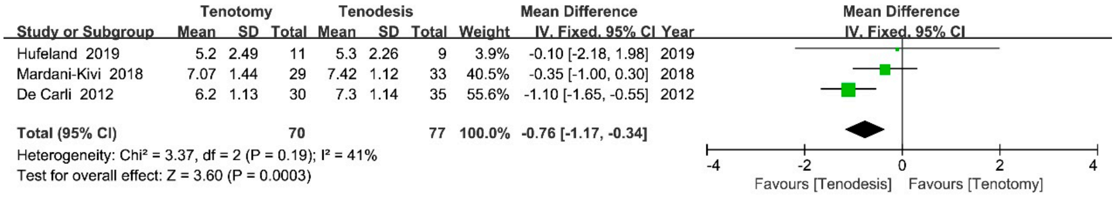

Figure S3A: meta-analysis results of ESI

Study or Subgroup	Tenotomy Mean	Tenotomy SD	Tenotomy Total	Tenodesis Mean	Tenodesis SD	Tenodesis Total	Weight	Mean Difference IV, Fixed, 95% CI	Year
Van Deurzen 2021	1.01	0.395	44	0.97	0.225	42	2.2%	0.04 [-0.10, 0.18]	2021
Hufeland 2019	0.86	0.447	11	0.951	0.334	9	0.3%	-0.09 [-0.43, 0.25]	2019
Lee 2016	0.966	0.078	56	0.956	0.032	72	85.0%	0.01 [-0.01, 0.03]	2016
Oh 2016	1.042	0.49	27	1.103	0.36	31	0.8%	-0.06 [-0.29, 0.16]	2016
Zhang 2015	0.9	0.2	77	0.9	0.2	74	9.8%	0.00 [-0.06, 0.06]	2015
De Carli 2012	0.9245	0.226	30	0.907	0.37	35	1.9%	0.02 [-0.13, 0.16]	2012
**Total (95% CI)**			**245**			**263**	**100.0%**	**0.01 [-0.01, 0.03]**	

Heterogeneity: Chi² = 1.00, df = 5 (P = 0.96); I² = 0%
Test for overall effect: Z = 0.87 (P = 0.38)

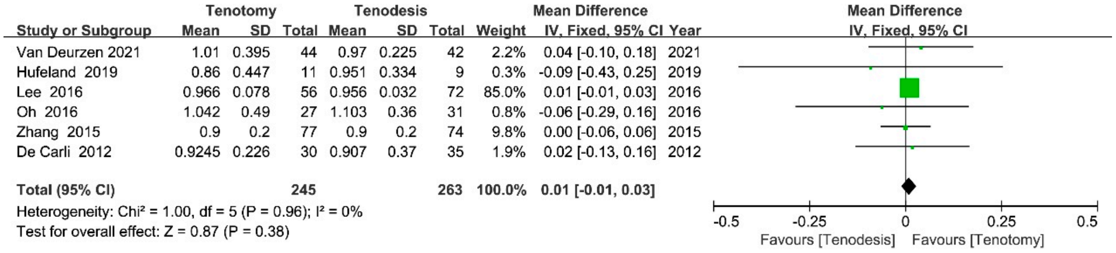

Figure S3B: meta-analysis results of FSSI

Study or Subgroup	Tenotomy Mean	Tenotomy SD	Tenotomy Total	Tenodesis Mean	Tenodesis SD	Tenodesis Total	Weight	Mean Difference IV, Random, 95% CI
Hufeland 2019	1.098	0.443	11	0.991	0.389	9	13.4%	0.11 [-0.26, 0.47]
Lee 2016	0.818	0.108	56	0.998	0.015	72	33.9%	-0.18 [-0.21, -0.15]
Oh 2016	1.29	0.53	27	0.87	0.44	31	19.5%	0.42 [0.17, 0.67]
Zhang 2015	0.9	0.2	77	0.9	0.1	74	33.2%	0.00 [-0.05, 0.05]
**Total (95% CI)**			**171**			**186**	**100.0%**	**0.04 [-0.14, 0.21]**

Heterogeneity: Tau² = 0.02; Chi² = 57.43, df = 3 (P < 0.00001); I² = 95%
Test for overall effect: Z = 0.41 (P = 0.68)

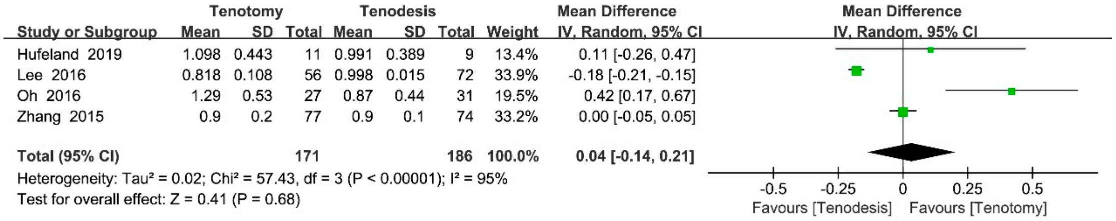

Figure S3C: meta-analysis results of ROM

Study or Subgroup	Tenotomy Mean	Tenotomy SD	Tenotomy Total	Tenodesis Mean	Tenodesis SD	Tenodesis Total	Weight	Mean Difference IV, Fixed, 95% CI	Year
Van Deurzen 2021	60.8	19.74	44	63.6	17.93	44	22.1%	-2.80 [-10.68, 5.08]	2021
Castricini 2018	47.1	10.5	31	49.4	14.7	24	28.5%	-2.30 [-9.25, 4.65]	2018
Oh 2016	45.19	8.263	27	44.52	12.07	31	49.4%	0.67 [-4.60, 5.94]	2016
**Total (95% CI)**			**102**			**99**	**100.0%**	**-0.94 [-4.65, 2.76]**	

Heterogeneity: Chi² = 0.72, df = 2 (P = 0.70); I² = 0%
Test for overall effect: Z = 0.50 (P = 0.62)

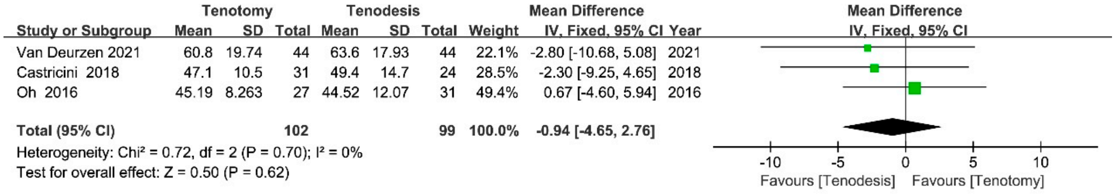

Supplement: Supplementary file 1 [file jcm-12-01754-s001.zip › supplementary figures.pdf]
